# HALP score-adjusted FRAXplus tool effectively predicts hip fracture risk in elderly women with type 2 diabetes mellitus

**DOI:** 10.1186/s12902-026-02338-3

**Published:** 2026-06-03

**Authors:** Wenxin Chu, Minghui Wu, Jingxia Sun, Jiangmei Pan, Zhengming Li, Jianhao Huang, Zhenwei Zhai, Wensheng Lu

**Affiliations:** 1 Department of Endocrinology and Metabolism, National Key Endocrine Clinical Construction Specialty, Guangxi Academy of Medical Sciences and the People’s Hospital of Guangxi Zhuang Autonomous Region, No. 6, Taoyuan Road, Nanning, Guangxi 530021 People’s Republic of China; 2 Department of Infectious Diseases, Guangxi Academy of Medical Sciences and the People’s Hospital of Guangxi Zhuang Autonomous Region, Nanning, Guangxi 530021 People’s Republic of China

**Keywords:** Elderly females, Type 2 diabetes mellitus, Fragility fracture, Fracture risk assessment tool, Hemoglobin, albumin, lymphocyte, and platelet score

## Abstract

**Background:**

Elderly women with type 2 diabetes mellitus (T2DM) are at high risk of fragility fractures, particularly lethal hip fractures. Pathophysiologically, this elevated risk may be attributed to reduced nutritional reserves and dysregulation of the immune-inflammatory system. However, the fracture risk assessment tool (FRAX) often underestimates the 10-year probability of future fractures, as it does not fully account for these pathophysiological factors in this high-risk population. This study aims to determine whether the hemoglobin, albumin, lymphocyte, and platelet (HALP) score serves as an effective prognostic marker for a real-world fragility fracture endpoint, and whether incorporating the HALP score into FRAX improves the prediction of the 10-year probability of fragility fractures in this high-risk population.

**Methods:**

This ambispective longitudinal cohort study utilized multicenter retrospective cohort data collected between January 1, 2014, and January 1, 2016 (Phase 1), followed by prospective follow-up for fragility fracture endpoints from January 1, 2016, to January 1, 2021 (Phase 2). Follow-up was conducted via outpatient services and telephone interviews every six months, with a median follow-up duration of 46.8 months. A total of 423 participants were included in the present analysis. Based on HALP score tertiles, participants were divided into low (≤ 31.98, *n* = 141), moderate (31.98–48.08, *n* = 140), and high (≥ 48.08, *n* = 142) groups.

**Results:**

Among the 423 participants, 75 (17.73%) experienced real-world fragility fractures. Spearman’s partial correlation analysis revealed a significant inverse association between the HALP score and the FRAX‑estimated 10‑year probability of hip fracture (FRAX‑HF) (*r* = − 0.103, *p* = 0.037), but no significant correlations with bone mineral density (BMD) and the FRAX‑estimated 10‑year probability of major osteoporotic fracture (FRAX‑MOF). Multivariate linear regression further quantified that each one‑tertile decrease in HALP (tertile interval = 16.1) was associated with an approximate 0.31% increase in FRAX‑HF probability (calculated as 0.019 × 16.1 = 0.3059) (β = −0.019; 95% confidence interval [CI]: −0.034 to − 0.005; *p* = 0.007). Notably, in the lowest HALP tertile, the FRAX‑HF increased from the overall baseline of 2.30% (moderate risk, requiring only lifestyle management) to 3.00% (high risk, warranting anti‑osteoporosis therapy), representing a relative increase of 30.43%. Consequently, all non‑osteoporotic participants in this tertile, whose initial hip fracture probability (2.3%) was below the guideline-recommended intervention threshold of 3%, met criteria for initiating anti‑osteoporosis treatment, resulting in an intervention rate of 27.13% (35/129). These findings support the utility of combining the HALP score with FRAX for risk stratification to refine hip fracture prediction. Restricted cubic spline (RCS) analysis indicated dose‑dependent nonlinear relationships between HALP and overall fragility fracture endpoint events (Z‑shaped, overall *p* = 0.0011), as well as between HALP and FRAX‑HF (L‑shaped, overall *p* < 0.001). Multivariable Cox regression confirmed a significant inverse trend between HALP and the real‑world fragility fracture endpoint (*p* for trend = 0.002), and subgroup stratified analyses demonstrated stable predictive value across strata (all *p* for interaction > 0.05). Kaplan–Meier analysis illustrated a higher cumulative fracture incidence associated with lower HALP scores (log‑rank test, *p* = 0.00037). Receiver operating characteristic (ROC) curve analysis identified an optimal HALP cut‑off of 46.77 for fragility fractures, with an area under the curve (AUC) of 0.643 and a sensitivity of 84.0% (*p* < 0.001).

**Conclusion:**

Our findings suggest that the HALP score may serve as an effective prognostic indicator for fragility fractures. Furthermore, the HALP score-adjusted FRAXplus tool (integrating the HALP score into FRAX) makes more elderly female patients with T2DM whose initial hip fracture probability falls below the guideline-recommended intervention threshold eligible for anti-osteoporosis therapy, thereby helping to reduce future 10-year hip fracture events.

**Clinical trial number:**

Not applicable.

**Supplementary Information:**

The online version contains supplementary material available at 10.1186/s12902-026-02338-3.

## Introduction

Driven predominantly by population aging, the global prevalence of type 2 diabetes mellitus (T2DM) and osteoporosis has risen to concerning levels. According to the recent International Diabetes Federation Atlas, the global diabetes burden has reached a staggering 537 million adults, with T2DM accounting for the overwhelming majority [1]. In parallel, approximately 200 million people suffer from osteoporosis, which contributes to as many as 9 million fragility fractures annually [2]. China mirrors this trend, with an adult diabetes prevalence of 12.8% [3] and age-standardized osteoporosis rates of 6.46% in men and 29.13% in women aged 50 years and older [4]. An analysis of the Chinese basic medical insurance database (2013–2017) reveals that 68.5% of vertebral fracture cases occurred in elderly women, with a mean age of 70.26 years [5]. This dual epidemic of metabolic and skeletal disorders poses a significant public health challenge in aging societies.

The fracture risk assessment tool (FRAX) has limitations in evaluating fracture risk in postmenopausal women with T2DM, as it does not adequately capture the roles of immunoinflammatory activity and malnutrition—key pathophysiological contributors to bone fragility in this population [6]. Previously, we explored the prognostic value of multiple indicators for predicting fragility fractures in postmenopausal women with T2DM, including the Geriatric Nutritional Risk Index (GNRI) [7], the systemic immune-inflammation index (SII) [8], and the systemic immune-inflammation index-to-albumin ratio (SAR) [9]. The present study aims to determine whether the hemoglobin, albumin, lymphocyte, and platelet (HALP) score serves as an effective prognostic marker for fragility fractures and whether it enhances the predictive performance of the FRAX-estimated 10-year probability of fragility fractures in this population.

The HALP score, derived from the formula (hemoglobin × albumin × lymphocytes) / platelets, is a recently proposed biomarker that can be readily calculated from routine blood test results [10]. Initially validated as a prognostic indicator in cancer research [11], its components reflect several critical pathophysiological pathways: hemoglobin depletion due to chronic inflammation [12], hypoalbuminemia linked to malnutrition-associated musculoskeletal loss [13], lymphopenia that impairs immunosurveillance mediated by the receptor activator of nuclear factor kappa B ligand (RANKL) [14], and thrombocytosis that promotes the release of inflammatory mediators [15]. In addition, platelet-derived growth factors enhance the chemotaxis of osteoclast precursors, thereby initiating bone loss [16]. Recent evidence confirms the HALP score’s predictive value for mortality among elderly patients with femoral fractures [17], supporting its potential application to diabetic bone disease. The HALP score objectively quantifies the inflammatory burden using lymphocyte and platelet counts and hemoglobin levels, and assesses nutritional status using hemoglobin and albumin levels. This makes it an ideal candidate metric (as a FRAXplus tool) for enhancing the FRAX assessment. Unlike the existing factor-adjusted FRAXplus developed by the FRAX team (https://fraxplus.org/frax-plus), this study proposes, for the first time, a novel fracture risk stratification that integrates the HALP score into FRAX.

## Materials and methods

### Study design and participant selection

**Fig. 1:**
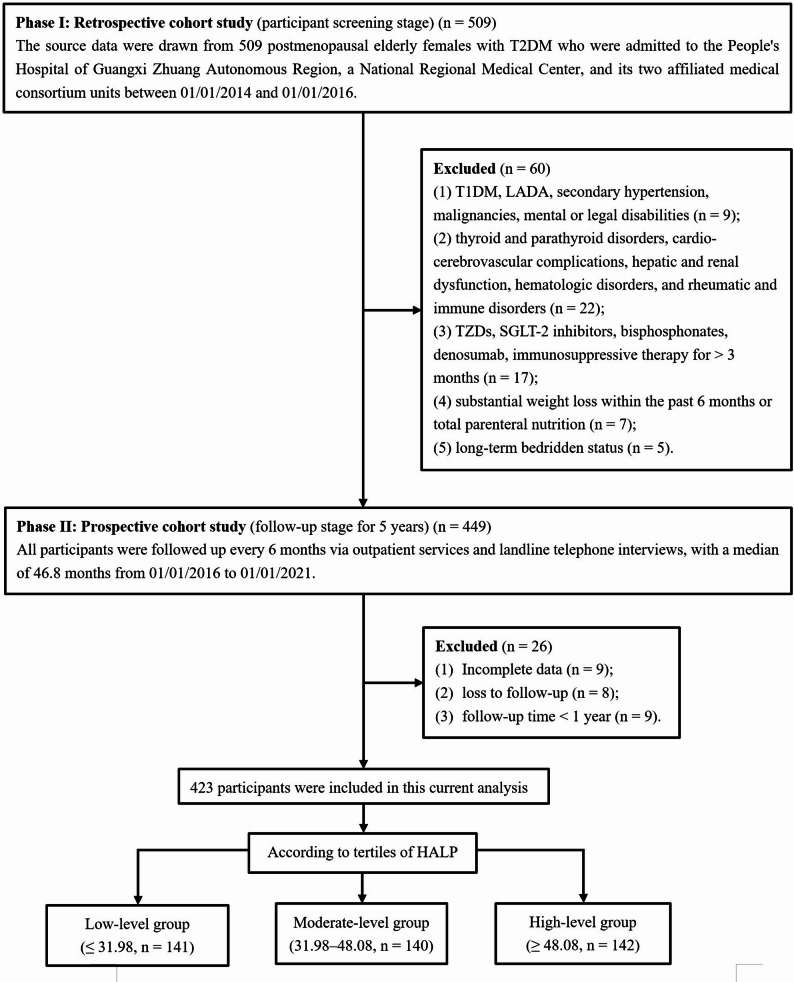
The flow chart for the participant selection strategy. This ambispective longitudinal cohort study used multicentre retrospective data collected between January 2014 and January 2016, with prospective follow-up for fragility fractures conducted between January 2016 and January 2021. Finally, 423 qualified participants were divided into low (≤ 31.98, *n* = 141), moderate (31.98–48.08, *n* = 140), and high (≥ 48.08, *n* = 142) groups based on HALP score tertiles

The participant selection strategy is illustrated in Fig. 1. This study was designed as an ambispective longitudinal cohort study (a retrospective cohort with prospective follow-up), consisting of two phases: Phase I—retrospective cohort establishment (participant screening over 2 years), and Phase II—prospective cohort follow-up (over 5 years). The source data were initially drawn from 509 postmenopausal elderly females with T2DM, who were admitted to the People’s Hospital of Guangxi Zhuang Autonomous Region (a National Regional Medical Center) and its two affiliated medical consortium units between January 1, 2014, and January 1, 2016. From this pool, 449 eligible participants were followed up every 6 months via outpatient services and landline telephone interviews from January 1, 2016, to January 1, 2021, with a median follow-up duration of 46.8 months. Thus, the maximum follow-up period was 7 years, encompassing retrospective record retrieval and prospective follow-up for real-world fragility fracture endpoint events. Ultimately, using a complete-case analysis method, 423 participants were included in the final analysis. Inclusion criteria were: postmenopausal women with a definitive T2DM diagnosis according to the 1999 World Health Organization (WHO) criteria [18], and availability of complete bone mineral density (BMD) data from dual-energy X-ray absorptiometry (DXA) as well as digital radiography (DR) imaging of the lumbar spine and pelvis. Exclusion criteria were as follows: (1) type 1 diabetes mellitus (T1DM), latent autoimmune diabetes in adults (LADA), secondary hypertension, malignancies, or mental or legal disabilities (*n* = 9); (2) thyroid or parathyroid disorders, cardio-cerebrovascular complications, hepatic or renal dysfunction, hematologic disorders, or rheumatic and immune disorders (*n* = 22); (3) use of thiazolidinediones (TZDs), sodium-glucose cotransporter 2 (SGLT-2) inhibitors, bisphosphonates, denosumab, or immunosuppressive therapy affecting bone turnover for more than 3 months (*n* = 17); (4) substantial weight loss within the past 6 months or receiving total parenteral nutrition (*n* = 7); (5) long-term bedridden status (*n* = 5); (6) incomplete data (*n* = 9), loss to follow-up (*n* = 8), or follow-up duration < 1 year (*n* = 9).

### Data collection

During the retrospective cohort establishment phase, all baseline data were extracted from the Hospital Information System (HIS), including demographic information (age, gender, ethnicity, occupation, and residence), lifestyle factors (smoking, alcohol consumption, and exercise habits), medical history, medication history (particularly for diabetes and osteoporosis), family genetic background, and blood biochemical indicators. The location, timing, and etiology of fragility fractures were confirmed using the Medical Record Information System, routine follow-up, and imaging data. The BMD measurements—at the lumbar spine (L1–L4), left femoral neck, and total hip—were obtained using a DXA scanner (Hologic, USA) operated by a technician certified by the Chinese Medical Association of Bone Mineral and Osteoporosis. The scanner was calibrated daily and underwent routine quality control. Each site was scanned three times, and the area BMD (aBMD) and T-scores were calculated. To ensure data integrity, key variables were independently entered by two staff members and cross-verified. During the prospective follow-up phase, participants were tracked every six months through outpatient visits, telephone calls, and linkage to the medical insurance reimbursement database, with a median follow-up duration of 46.8 months. Loss to follow-up was defined as no medical contact for 24 consecutive months, yielding an overall rate of 1.8% (8/449). All fragility fracture endpoint events were identified through retrospective medical record review and prospective follow-up, and confirmed by imaging (X-ray, computed tomography [CT], or magnetic resonance imaging [MRI]). Two orthopedic surgeons independently assessed these events, and a third expert resolved any discrepancies to minimize verification bias. In accordance with the complete-case analysis method, only participants with nearly complete datasets (missingness < 5%) were retained, while those with substantial missing data or lost to follow-up were excluded. Finally, a standardized research database was constructed for analysis, with all personally identifiable information anonymized to protect participant privacy.

### Definitions

This study adopted the following definitions: (1) Fragility fracture was defined as an endpoint event that required the fulfillment of all of the following criteria, each of which had to be independently confirmed by three orthopedic specialists: a clear fracture diagnosis supported by radiological evidence; fractures resulting from falls at standing height or lower; and the exclusion of high-impact traumatic fractures (e.g., caused by traffic accidents or falls from great heights) as well as pathological fractures. The first fracture was identified as the endpoint event among the multiple fractures observed during follow-up. Furthermore, fracture-free survival was defined as the time from retrospective enrollment to the occurrence of a fracture and was not considered a censoring event. (2) Patients who had no real-world fracture by the end of follow-up or who experienced a fracture within the first year of follow-up were censored at the earlier of the two events. (3) The baseline was defined as the most recent laboratory biochemical results obtained before discharge. (4) HALP score = [hemoglobin (g/L) × albumin (g/L) × lymphocyte counts (10^9^/L)]/platelet counts (10^9^/L). (5) The individualized 10-year probabilities of major osteoporotic fracture (MOF) and hip fracture (HF) were estimated using the FRAX (https://frax.shef.ac.uk/FRAX/tool.aspx?lang=chs). According to the China Guidelines for Diagnosis and Treatment of Primary Osteoporosis (2022), fragility fracture risk is classified using FRAX as follows: Low risk: FRAX-HF < 1.5% or FRAX-MOF < 10%; Moderate risk: FRAX-HF 1.5%–3.0% or FRAX-MOF 10%–20%; High risk: FRAX-HF 3.0%–4.5% or FRAX-MOF 20%–30%; Very high risk: FRAX-HF ≥ 4.5% or FRAX-MOF ≥ 30%. The guideline-recommended threshold for initiating a standardized anti-osteoporosis therapy is a FRAX-HF ≥ 3% or a FRAX-MOF ≥ 20%. (6) According to the Law of the People’s Republic of China on the Protection of the Rights and Interests of the Elderly, individuals aged 60 or above are legally defined as elderly persons.

### Statistical analyses

All statistical analyses were performed using SPSS 26.0 (IBM Corp., Armonk, NY, USA). All graphical representations were generated using R version 4.2.2 (R Foundation for Statistical Computing, Vienna, Austria), including automatically generated baseline tables produced with the tableone package. All data analyses were performed using complete-case analysis (CCA), multiple imputation with chained equations (MICE) to address missing data (< 5%), and 1,000 bootstrap resampling iterations to assess model robustness.

Continuous variables following a normal distribution were presented as means ± standard deviations (SD), whereas non-normally distributed variables were reported as medians with interquartile ranges (IQRs). Categorical variables were expressed as frequencies (percentages). For group comparisons, the independent-samples t-test was used for normally distributed continuous variables; the Kruskal–Wallis H test, with post hoc adjustments for multiple comparisons, was used for non-normally distributed continuous variables; and the chi-square test was used for categorical variables. When the Kruskal–Wallis H test yielded a statistically significant result (*p* < 0.05), post hoc pairwise comparisons were conducted using Dunn’s test with Bonferroni correction to control the overall Type I error rate. Spearman’s partial correlation analysis, multivariate linear regression, and restricted cubic spline (RCS) models were used to assess both linear and nonlinear relationships. Univariable and multivariable Cox proportional hazards regression models were performed to evaluate all prognostic risk factors. Subgroup-stratified analyses were conducted to examine potential confounders, with results presented as forest plots. Kaplan–Meier analysis was used to estimate the cumulative incidence of fragility fractures, and group differences were tested using the log-rank test. Receiver operating characteristic (ROC) curve analysis identified the optimal HALP score cutoff for predicting the risk of fragility fractures. A *p*-value < 0.05 was considered statistically significant in this study.

Additionally, we conducted a post-hoc power analysis using G*Power 3.1.9.2 (A priori module for one-way ANOVA). With a total sample size of 423 participants, a fracture incidence rate of 0.18 (75/423), grouping based on HALP score tertiles, and α = 0.05, the analysis indicated an actual power of 92%, which exceeds the recommended threshold of > 80% for clinical research (see Supplementary Material Fig. 1).

## Results

### Baseline characteristics

The baseline characteristics of the subjects are presented in Table 1. Based on HALP score tertiles, participants were divided into three groups: low (≤ 31.98, *n* = 141), moderate (31.98–48.08, *n* = 140), and high (≥ 48.08, *n* = 142). Significant differences were observed among the three groups for several variables, including hypertension, fracture, body mass index (BMI), neutrophil counts, free triiodothyronine (FT3), creatinine (Cr), calcium (Ca), and 25-hydroxy vitamin D [25-(OH) D] (all *p* < 0.05). Additionally, variables such as haemoglobin (Hb), albumin (ALB), lymphocytes, platelets, lumbar spine (LS)-BMD, femoral neck (FN)-BMD, total hip (TH)-BMD, FRAX-MOF, and FRAX-HF also differed significantly across the three HALP groups (all *p* < 0.05), suggesting an association between HALP scores and fragility fractures.

**Table 1:** Baseline characteristics

Characteristics	All participants (*n* = 423)	Low (HALP ≤ 31.98, *n* = 141)	Moderate (HALP 31.98–48.08, *n* = 140)	High (HALP ≥ 48.08, *n* = 142)	*p*-values
Age, years	69.00 (64.00–75.00)	71.00 (65.00–76.00)	69.00 (62.00-75.25)	68.00 (63.00–75.00)	0.062
Duration of diabetes, years	10.00 (3.00–16.00)	10.00 (3.00–14.00)	10.00 (3.00–20.00)	7.00 (2.00-14.75)	0.086
DPN, n (%)					0.870
No	158 (37.35%)	53 (37.59%)	50 (35.71%)	55 (38.73%)	
Yes	265 (62.65%)	88 (62.41%)	90 (64.29%)	87 (61.27%)	
PVD, n (%)					0.509
No	41 (9.69%)	17 (12.06%)	12 (8.57%)	12 (8.45%)	
Yes	382 (90.31%)	124 (87.94%)	128 (91.43%)	130 (91.55%)	
Hypertension, n (%)					0.018^*^
No	146 (34.52%)	41 (29.08%)	43 (30.71%)	62 (43.66%)	
Yes	277 (65.48%)	100 (70.92%)	97 (69.29%)	80 (56.34%)	
Osteoporosis, n (%)					0.107
No	129 (30.50%)	35 (24.82%)	51 (36.43%)	43 (30.28%)	
Yes	294 (69.50%)	106 (75.18%)	89 (63.57%)	99 (69.72%)	
Fracture history, n (%)					0.261
No	309 (73.05%)	96 (68.09%)	105 (75.00%)	108 (76.06%)	
Yes	114 (26.95%)	45 (31.91%)	35 (25.00%)	34 (23.94%)	
Fracture, n (%)					< 0.001^*^
No	348 (82.27%)	103 (73.05%)	115 (82.14%)	130 (91.55%)	
Yes	75 (17.73%)	38 (26.95%)	25 (17.86%)	12 (8.45%)	
BMI, kg/m^2^	24.24 (22.07–26.78)	23.78 (21.46–26.12)	24.73 (22.28–26.94)	24.31 (22.44-27.00)	0.046^*^
WBC, 10^9^/L	6.99 (5.89–8.48)	7.31 (5.74–9.05)	6.87 (5.71–7.95)	7.01 (6.16–8.43)	0.089
Neutrophil, 10^9^/L	4.16 (3.25–5.29)	4.92 (3.87–6.59)	3.88 (3.24–5.06)	3.78 (3.08–4.75)	< 0.001^*^
Platelet, 10^9^/L	241.00 (202.00-291.00)	274.00 (235.00-341.00)	247.50 (206.75-293.25)	209.50 (175.00-240.50)	< 0.001^*^
Lymphocyte, 10^9^/L	1.99 (1.56–2.46)	1.53 (1.27–1.85)	2.04 (1.68–2.33)	2.46 (2.17–2.99)	< 0.001^*^
Hb, g/L	125.00 (115.00-134.00)	115.00 (103.00-125.00)	126.00 (119.00-134.00)	131.50 (122.00-140.75)	< 0.001^*^
FBG, mmol/L	7.27 (5.64–9.61)	6.94 (5.46–8.78)	7.45 (5.77–9.47)	7.33 (5.59–10.15)	0.451
HbA1c, %	8.20 (6.90–10.40)	8.00 (6.80–10.20)	8.20 (6.90-10.22)	8.40 (7.10-10.88)	0.407
TSH, µIU/mL	1.68 (1.08–2.61)	1.71 (1.08–2.66)	1.47 (1.01–2.37)	1.82 (1.28–2.64)	0.062
FT3, pmol/L	4.50 (4.09–4.92)	4.37 (4.00-4.76)	4.58 (4.15–4.98)	4.53 (4.17–5.05)	0.017^*^
FT4, pmol/L	11.25 (9.86–13.20)	11.71 (9.75–13.33)	11.38 (9.96–13.36)	10.79 (9.90-12.41)	0.099
TC, mmol/L	4.89 (4.00-5.75)	4.84 (3.90–5.76)	4.85 (4.09–5.74)	5.07 (4.03–5.71)	0.839
TG, mmol/L	1.42 (1.04–2.04)	1.34 (0.96–1.95)	1.47 (1.08–1.94)	1.48 (1.08–2.44)	0.117
HDL-C, mmol/L	1.17 (1.01–1.38)	1.16 (1.01–1.36)	1.17 (1.00-1.38)	1.17 (1.02–1.42)	0.880
LDL-C, mmol/L	2.92 (2.27–3.59)	2.88 (2.24–3.58)	2.92 (2.29–3.47)	2.92 (2.27–3.62)	0.829
Cr, µmol/L	65.00 (55.00–80.00)	70.00 (54.00–95.00)	61.50 (55.00–75.00)	65.00 (54.25–73.75)	0.013^*^
UA, µmol/L	317.00 (267.50–390.00)	317.00 (259.00-375.00)	320.50 (262.50-392.75)	316.50 (281.25-393.25)	0.820
Ca, mmol/L	2.27 (2.20–2.36)	2.23 (2.15–2.31)	2.28 (2.22–2.37)	2.29 (2.23–2.37)	< 0.001^*^
25-(OH) D, nmol/L	54.19 (39.22–67.58)	49.98 (32.53–61.58)	55.94 (43.02–68.94)	57.23 (42.17–72.81)	< 0.001^*^
ALB, g/L	38.40 (35.35-41.00)	34.50 (31.80–38.40)	39.20 (36.48–41.62)	39.50 (37.73–42.03)	< 0.001^*^
LS BMD, g/cm^2^	0.74 (0.66–0.84)	0.73 (0.66–0.81)	0.78 (0.67–0.87)	0.73 (0.64–0.83)	0.025^*^
FN BMD, g/cm^2^	0.56 (0.49–0.63)	0.54 (0.47–0.62)	0.56 (0.50–0.63)	0.58 (0.51–0.65)	0.046^*^
TH BMD, g/cm^2^	0.70 ± 0.13	0.67 ± 0.12	0.72 ± 0.14	0.72 ± 0.13	0.001^*^
FRAX-MOF, %	6.10 (4.10–9.60)	7.30 (4.40–12.00)	6.00 (3.98–9.22)	5.80 (4.10–8.95)	0.040^*^
FRAX-HF, %	2.30 (1.10–4.40)	3.00 (1.30–5.70)	2.20 (1.00-3.82)	2.15 (1.10-4.00)	0.028^*^

Notes: Mean ± standard deviation (SD) and median (Inter Quartile Range, IQR) for continuous variables. Percentage (%) for categorical variables. ^*^p < 0.05

Abbreviations: HALP, hemoglobin, albumin, lymphocyte, and platelet; DPN, diabetic peripheral neuropathy; PVD, peripheral vascular disease; BMI, body mass index; WBC, white blood cell counts; Hb, haemoglobin; FBG, fasting blood glucose; HbA1c, glycated haemoglobin; TSH, thyroid stimulating hormone; FT3, free triiodothyronine; FT4, free thyroxine; TC, total cholesterol; TG, triglycerides; HDL-C, high-density lipoprotein cholesterol; LDL-C, low-density lipoprotein cholesterol; Cr, creatinine; UA, uric acid; Ca, calcium; 25(OH) D, 25-hydroxy vitamin D; ALB, albumin; BMD, bone mineral density; LS, lumbar spine; FN, femoral neck; TH, total hip; FRAX, fracture risk assessment tool; MOF, major osteoporotic fracture; HF, hip fracture

According to the 2022 Chinese Guidelines for Diagnosis and Treatment of Primary Osteoporosis, standard anti-osteoporosis therapy is recommended for patients diagnosed with osteoporosis or with an FRAX-estimated HF probability of ≥ 3% (the guideline-defined intervention threshold). Among the 423 participants, 294 were diagnosed with osteoporosis. At the same time, the remaining 129 non-osteoporotic individuals, with a median HF probability of 2.30% (IQR: 1.10–4.40), were initially advised to pursue lifestyle management only, reflecting a moderate fracture risk below the intervention threshold. However, after integrating the HALP score into FRAXplus, 35 of these 129 non-osteoporotic participants (those in the low HALP tertile group) showed a median HF probability of 3.00% (IQR: 1.30–5.70), which exceeded the intervention threshold and thus met the criteria for standard anti-osteoporosis therapy (indicating high fracture risk). Notably, the HALP-adjusted FRAXplus tool increased the estimated hip fracture risk from 2.30% to 3.00%, representing a relative increase of 30.43% {[(3.00–2.30)/2.30] × 100%}. Consequently, all 35 non-osteoporotic participants in this tertile, whose initial HF probability (2.3%) fell below the 3% intervention threshold, now qualify for anti-osteoporosis treatment, corresponding to an intervention rate of 27.13% (35/129). These findings support the value of combining HALP with FRAX to refine hip fracture risk stratification.

### Spearman’s partial correlation analysis

The linear correlation analysis between the HALP and BMD, as well as FRAX-estimated 10-year probabilities of MOF and HF, is presented in Table 2. After adjusting for confounding factors, such as age, hypertension, fracture history, peripheral vascular disease (PVD), BMI, neutrophil, FT3, free thyroxine (FT4), Ca, Cr, and triglycerides (TG) in the model III, Spearman’s partial correlation analysis showed a significant negative association between HALP and FRAX-estimated 10-year probability of HF (*r* = -0.103, *p* = 0.037), but no association with LS-BMD (*r* = 0.015, *p* = 0.768), FN-BMD (*r* = 0.055, *P* = 0.265), TH-BMD (*r* = 0.081, *p* = 0.102), and MOF (*r* = -0.093, *p* = 0.058). These findings indicate that the HALP serves as an adjustment factor for FRAX, enhancing the efficacy of HF probability assessment.

**Table 2:** Spearman’s partial correlation analysis for the linear correlations between the HALP score and BMDs, and the individualized 10-year probability of MOF and HF estimated by FRAX

	LS-BMD		FN-BMD		TH-BMD		FRAX-MOF		FRAX-HF	
	*r*	*p*-values	*r*	*p*-values	*r*	*p*-values	*r*	*p*-values	*r*	*p*-values
Model I	0.061	0.208	0.153	0.002^*^	0.186	<0.001^*^	-0.157	0.001^*^	-0.161	<0.001^*^
Model II	0.057	0.242	0.132	0.007^*^	0.157	0.001^*^	-0.137	0.005^*^	-0.144	0.003^*^
Model III	0.015	0.768	0.055	0.265	0.081	0.102	-0.093	0.058	-0.103	0.037^*^

Notes: Model I: adjusted for none. Model II: adjusted for hypertension and fracture history. Model III: adjusted for age, hypertension, fracture history, PVD, BMI, Neutrophil, FT3, FT4, Ca, Cr, and TG. ^*^ *p* < 0.05

Abbreviations: HALP, hemoglobin, albumin, lymphocyte, and platelet; BMD, bone mineral density; MOF, major osteoporotic fracture; HF, hip fracture; FRAX, fracture risk assessment tool; LS, lumbar spine; FN, femoral neck; TH, total hip; PVD, peripheral vascular disease; BMI, body mass index; FT3, free triiodothyronine; FT4, free thyroxine; Ca, calcium; Cr, creatinine; TG, triglycerides

### Multivariate linear regression analysis

Although FN-BMD, TH-BMD, and FRAX-MOF yielded significant p-values in model III of the multivariate linear regression analysis (Table 3), the overall relationship was nonlinear. Therefore, we focus our discussion solely on the quantitative impact of FRAX-HF, which showed a statistically significant negative correlation, as confirmed by Spearman’s partial correlation analysis. Multivariate linear regression further revealed that each one‑tertile decrease in HALP (tertile interval = 16.1) was associated with an approximate 0.31% increase in FRAX-HF probability (calculated as 0.019 × 16.1 = 0.3059) (β = −0.019; 95% confidence interval [CI]: −0.034 to − 0.005; *p* = 0.007). Notably, as shown in Table 1, in the lowest HALP tertile, FRAX-HF increased from the overall baseline of 2.30% (moderate risk, requiring only lifestyle management) to 3.00% (high risk, warranting anti-osteoporosis therapy), representing a relative increase of 30.43%. These findings support the utility of combining HALP with FRAX to refine hip fracture risk prediction, enabling more elderly T2DM females whose initial hip fracture probability falls below the guideline-recommended intervention threshold to become eligible for anti-osteoporosis therapy, thereby potentially reducing future 10-year hip fracture events. Although this analysis offers a novel perspective, it should not be overinterpreted and should serve only as one of several bases for fracture risk stratification in routine clinical practice.

**Table 3:** Multivariate linear regression analysis for the association between HALP score and osteoporosis-related indices

Indices	Model I β (95% CI)	*p*-values	Model II β (95% CI)	*p*-values	Model III β (95% CI)	*p*-values
FRAX-HF, %	-0.029 (-0.045, -0.013)	0.001^*^	-0.024 (-0.040, -0.008)	0.004^*^	-0.019 (-0.034, -0.005)	0.007^*^
FN-BMD, g/cm²	0.001 (0.000, 0.001)	0.002^*^	0.001 (0.000, 0.001)	0.012^*^	0.001 (0.000, 0.001)	0.022^*^
TH-BMD, g/cm²	0.001 (0.001, 0.002)	< 0.001^*^	0.001 (0.000, 0.001)	0.003^*^	0.001 (0.000, 0.001)	0.005^*^
FRAX-MOF, %	-0.039 (-0.062, -0.016)	0.001^*^	-0.030 (-0.051, -0.008)	0.008^*^	-0.022 (-0.040, -0.005)	0.012^*^
LS-BMD, g/cm²	0.000 (-0.000, 0.001)	0.145	0.000 (-0.000, 0.001)	0.23	0.000 (-0.000, 0.001)	0.356

Notes: Model I: adjusted for none. Model II: adjusted for age and duration of diabetes. Model III: adjusted for age, duration of diabetes, and fracture history. ^*^ *p* < 0.05

Abbreviations: HALP, hemoglobin, albumin, lymphocyte, and platelet; FRAX, fracture risk assessment tool; HF, hip fracture; FN, femoral neck; BMD, bone mineral density; TH, total hip; MOF, major osteoporotic fracture; LS, lumbar spine

### Restricted cubic spline model analysis

**Fig. 2:**
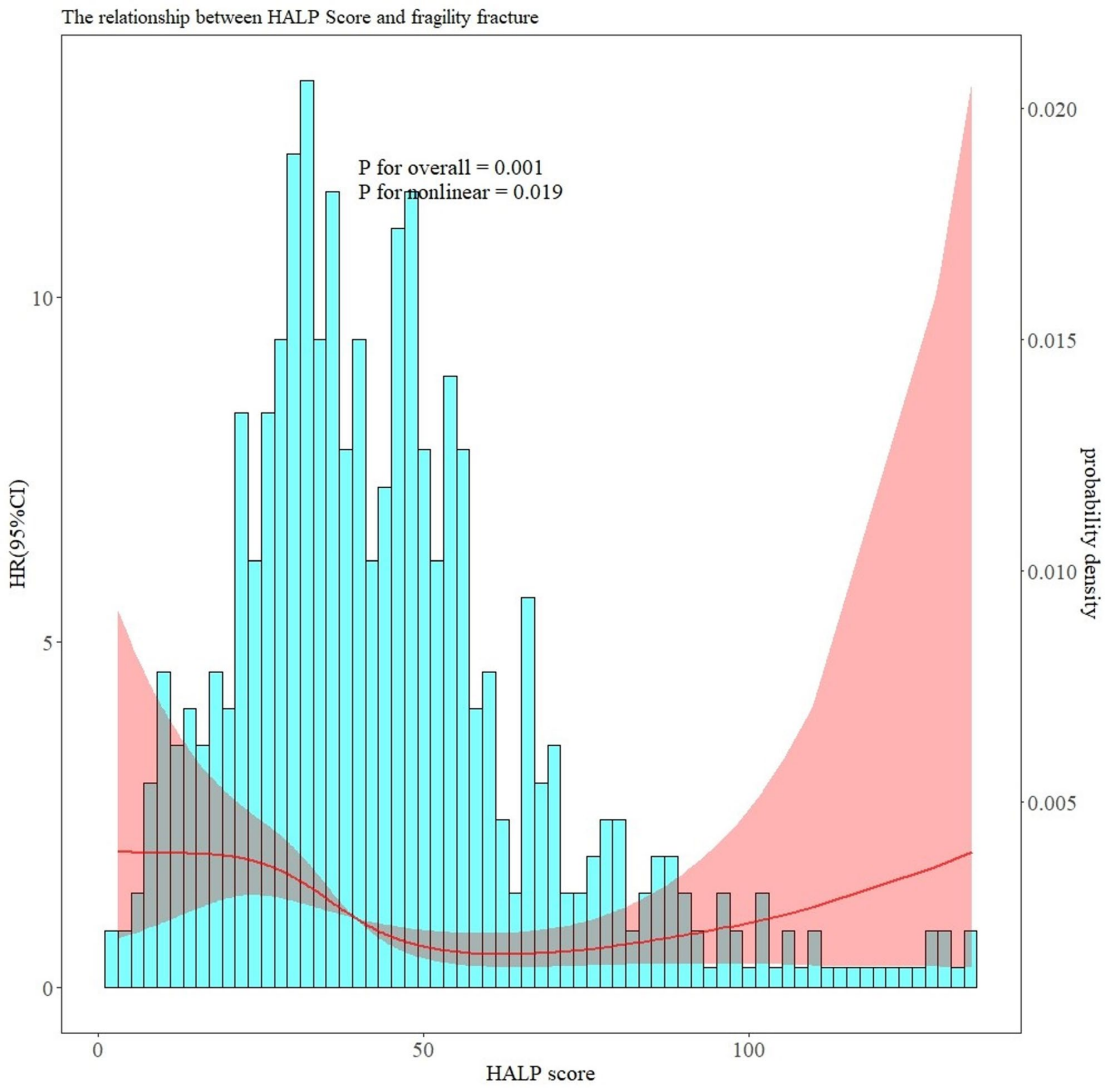
The nonlinear relationship between the HALP score and the risk of fragility fractures. The restricted cubic spline (RCS) models assessed the association between the HALP score and a real-world fragility fracture endpoint event. RCS analysis revealed a Z-shaped, dose-dependent correlation between HALP and a fracture endpoint (overall *p* = 0.001; nonlinear *p* = 0.019). The blue bars represent the distribution of case numbers

**Fig. 3:**
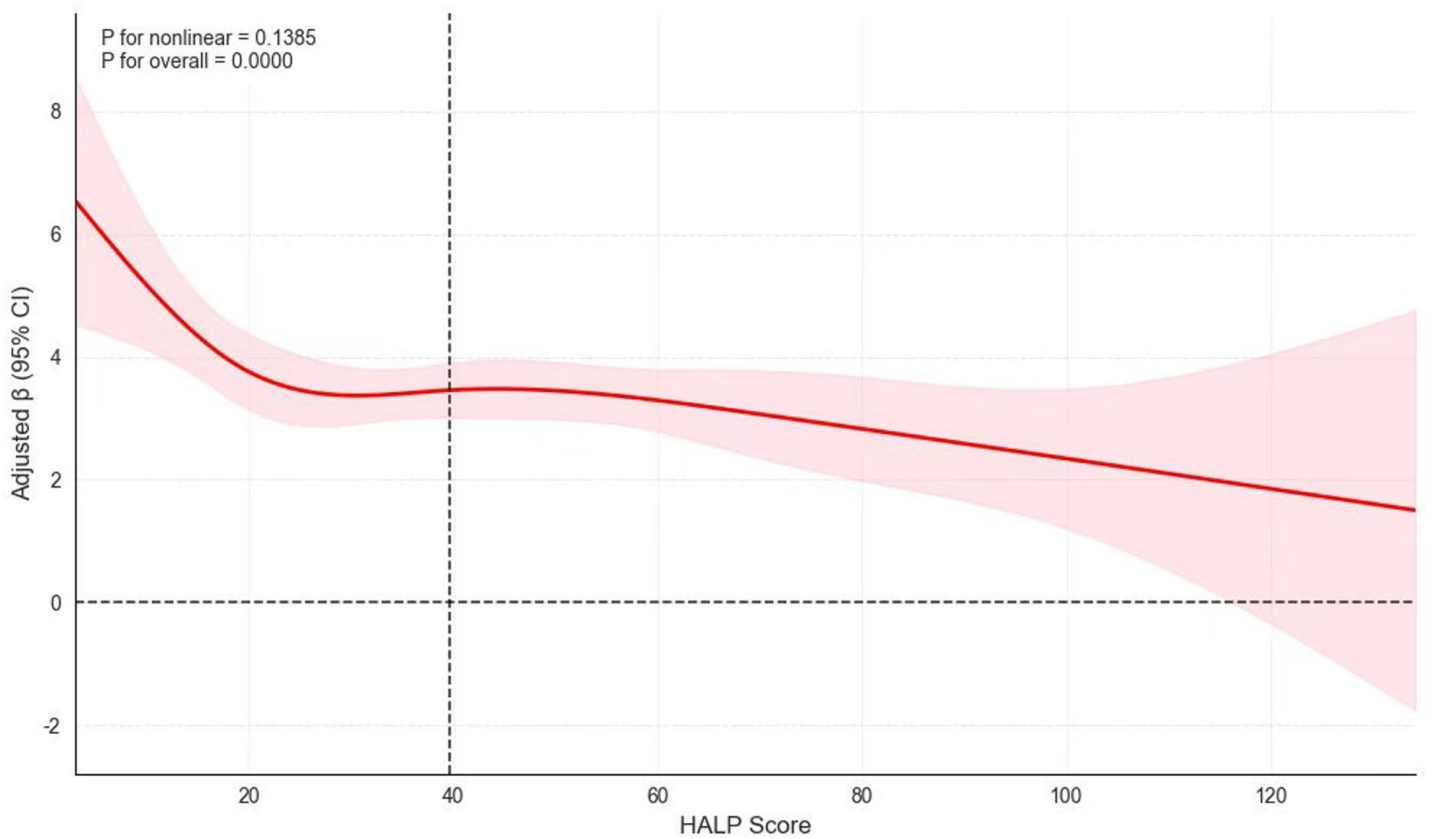
The nonlinear relationship between the HALP score and FRAX-estimated hip fracture probability. The restricted cubic spline (RCS) models assessed the association between the HALP score and the FRAX-estimated 10-year probability of hip fracture (FRAX-HF) using Spearman partial correlation analysis data in model III. RCS analysis showed an L-shaped, dose-dependent correlation between HALP and FRAX-HF (overall *p* < 0.001)

The dose-response relationships between the HALP score and fragility fracture outcomes are presented in Figs. 2 and 3. Using RCS models based on Spearman partial correlation data from Model III, we evaluated the association of HALP with a real-world fragility fracture endpoint (Fig. 2) and with the FRAX-estimated 10-year probability of hip fracture (Fig. 3), after adjusting for age, hypertension, fracture history, peripheral vascular disease (PVD), body mass index (BMI), neutrophil count, FT3, free thyroxine (FT4), Ca, Cr, and triglycerides (TG). Four knots were placed at the 5th, 35th, 65th, and 95th percentiles of the HALP distribution, with the 5th percentile set as the reference. The RCS analysis revealed a Z-shaped, dose-dependent relationship between HALP and fragility fracture risk (*p*-overall = 0.001, *p*-nonlinear = 0.019; Fig. 2), with the solid line representing hazard ratios (HRs) and the shaded area denoting 95% confidence intervals (CIs). Furthermore, an L-shaped dose-dependent correlation was observed between HALP and the FRAX-estimated 10-year hip fracture probability (*p*-overall = 0.002; Fig. 3), with the solid line indicating regression coefficients (β) and the shading representing 95% CIs. Collectively, these analyses demonstrate that higher HALP scores are associated with a reduced risk of fragility fractures and a lower 10-year probability of hip fracture, underscoring HALP’s critical role in predicting hip fracture risk.

### Multivariable Cox proportional hazards regression analysis

The results of the multivariable Cox proportional hazards regression analysis are presented in Table 4. The analysis revealed an inverse association between the HALP score and a real-world fragility fracture endpoint (*p* for trend = 0.002). Specifically, after full adjustment for confounding variables in Model III, multivariable Cox regression analysis showed that the risk of fragility fracture in the low-level group was significantly higher than that in the high-level group (HR = 2.73, 95% CI = 1.42–5.25, *p* = 0.003). This indicates that the HALP score is a negative predictor of fracture risk.

**Table 4:** Multivariable Cox regression analysis for the relationship between the HALP score and a real-world fragility fracture endpoint

Groups	Case/total	HR (95% CI)					
		Model I	*p*-values	Model II	*p*-values	Model III	*p*-values
**All participants**	75/423	0.98 (0.97–0.99)	0.001^*^	0.98 (0.97-1.00)	0.008^*^	0.98(0.97-1.00)	0.017^*^
**Low** (≤ 31.98)	38/141	3.48 (1.82–6.66)	< 0.001^*^	2.94 (1.53–5.56)	0.001^*^	2.73 (1.42–5.25)	0.003^*^
**Moderate** (31.98–48.08)	25/140	2.35(1.18–4.67)	0.015^*^	2.22 (1.11–4.45)	0.024^*^	1.97 (0.98–3.96)	0.057
**High** (≥ 48.08)	12/142	Reference		Reference		Reference	
**P for trend**			< 0.001*		0.001*		0.002*

Notes: Model I: adjusted for none. Model II: adjusted for age and duration of diabetes. Model III: adjusted for age, duration of diabetes, and fracture history. * *p* < 0.05

Abbreviations: HALP, hemoglobin, albumin, lymphocyte, and platelet; HR, hazard ratio; CI, confidence interval

### Stratified analysis in subgroups

**Fig. 4:**
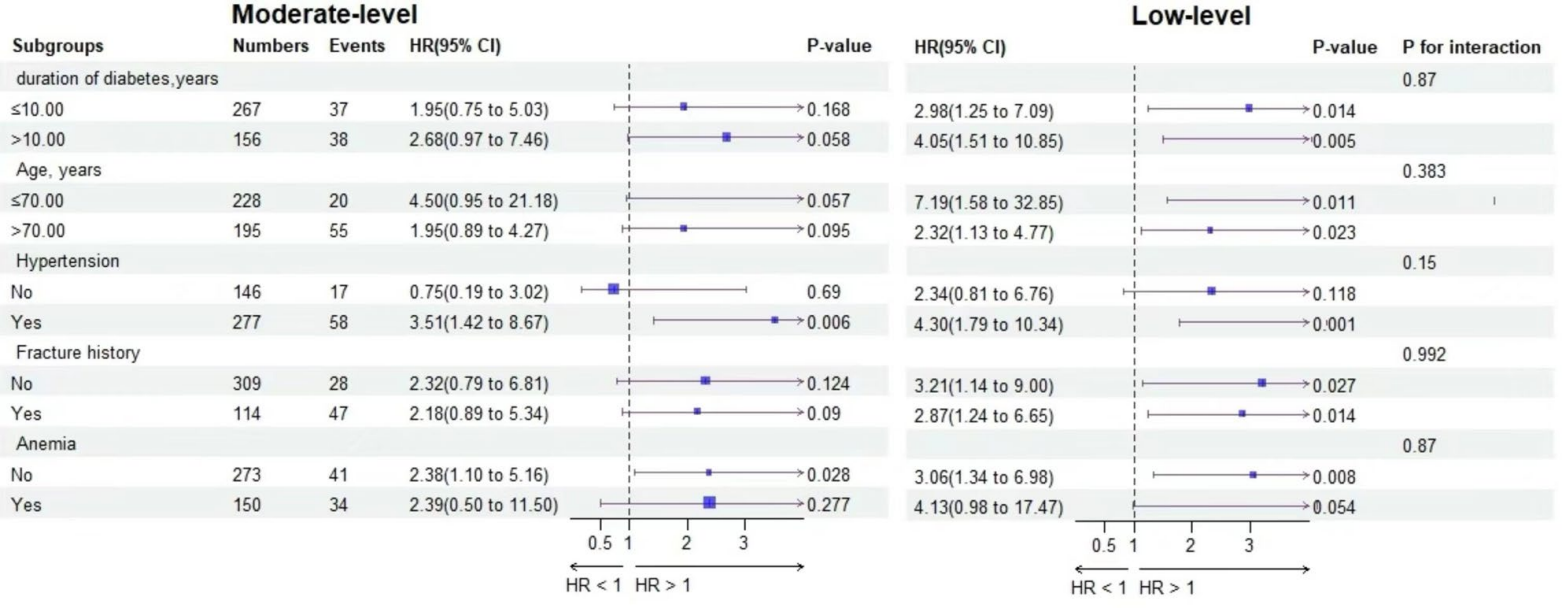
The forest plot for stratified subgroup analysis. Stratified analysis revealed that stratification factors did not affect the association between the HALP scores and real-world fragility fractures across the entire study population (*p* for interaction > 0.05)

The forest plot in Fig. 4 presents the results of the stratified subgroup analyses, which demonstrated that the association between the HALP score and fragility fractures remained consistent across the entire study population, regardless of stratification by diabetes duration, age, hypertension, fracture history, or anemia (all *p* for interaction > 0.05). This consistency indicates that the predictive value of the HALP score is stable across different subgroups, further underscoring its effectiveness as an independent predictor of fragility fracture risk.

### Kaplan–Meier survival analysis

**Fig. 5:**
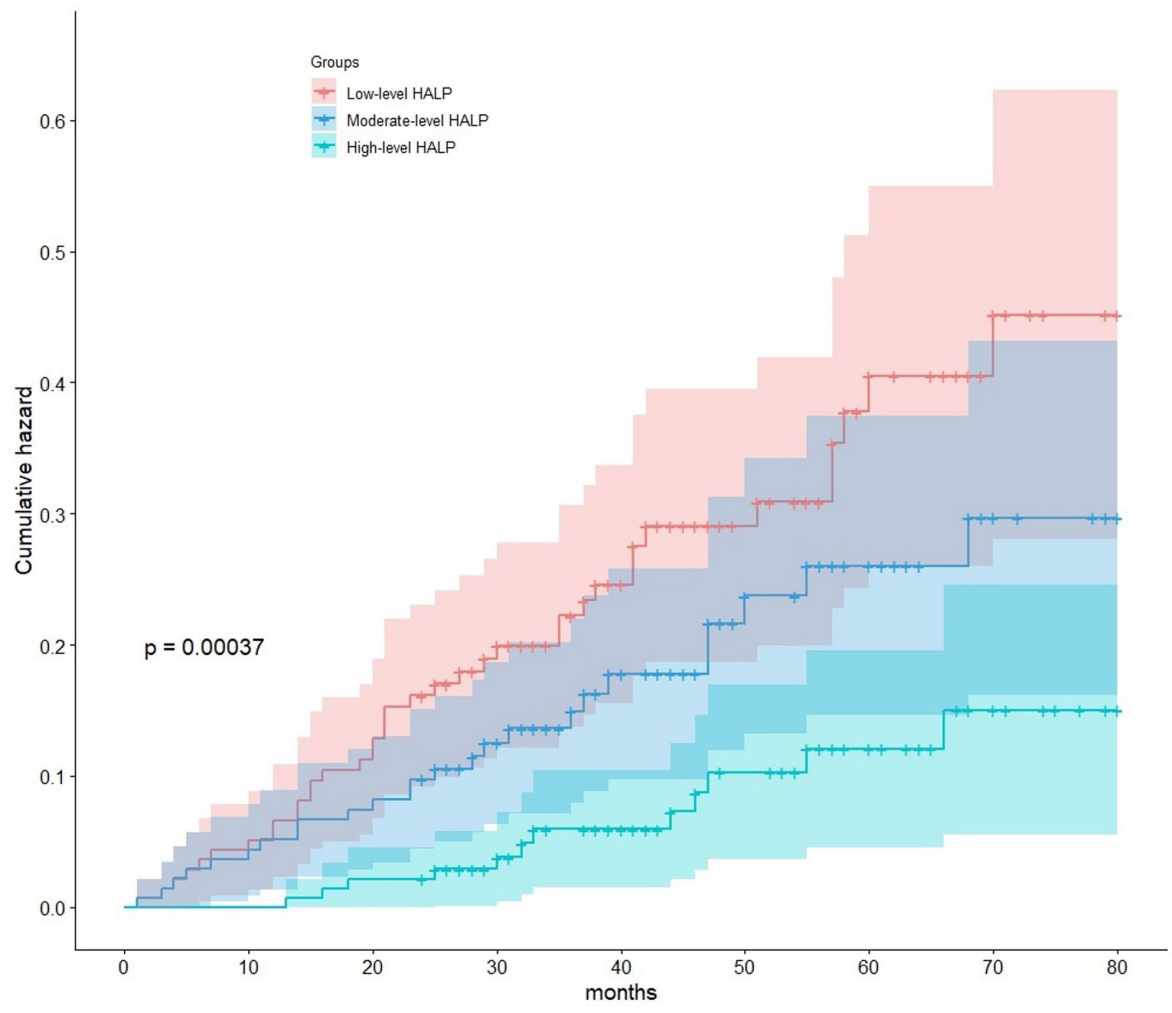
Kaplan–Meier survival analysis for the cumulative incidence of fragility fractures. The Kaplan-Meier survival curves demonstrated that lower HALP levels were associated with a higher cumulative incidence of real-world fragility fractures (log-rank, *p* = 0.00037)

The Kaplan-Meier survival curve is presented in Fig. 5. Among the three groups stratified by different HALP scores, the analysis revealed that lower HALP levels were associated with a higher cumulative incidence of real-world fractures (log-rank test, *p* = 0.00037). This finding confirms the prognostic value of the HALP score for fragility fractures.

### Receiver operating characteristic analysis

**Fig. 6:**
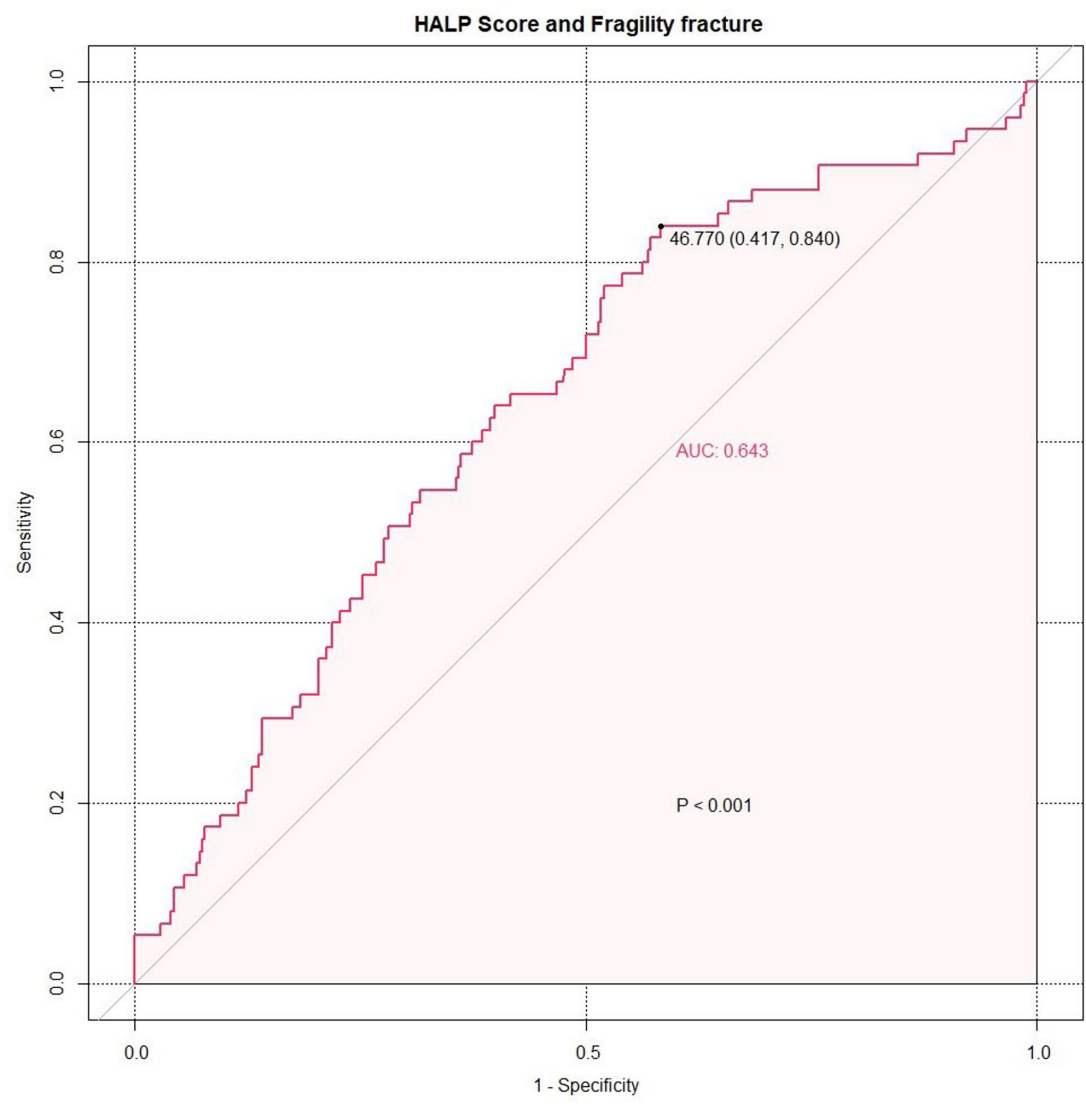
The diagnostic efficacy of the HALP score for fragility fractures. The receiver operating characteristic (ROC) curve analysis identified the HALP optimal cutoff value of 46.77, with an area under the curve (AUC) of 0.643, a sensitivity of 84.0%, and a specificity of 41.7%

The ROC curve is shown in Fig. 6. To guide routine clinical practice, an ROC analysis was performed to evaluate the prognostic value of the HALP score for the risk of fragility fracture. Using Youden’s index (0.257), the optimal HALP cut-off value for predicting fragility fracture was determined to be 46.77, corresponding to an area under the curve (AUC) of 0.643 (95% CI = 0.589–0.697, *p* < 0.001), a sensitivity of 84.0%, and a specificity of 41.7%. These findings suggest that the HALP score may serve as a potential predictor of fragility fracture risk in elderly females with T2DM.

### Missing data handling, sensitivity analysis, and internal validation

#### Missing data handling and sensitivity analysis

Missing categorical variables were labeled as “unknown” and included as dummy variables in subsequent models. For continuous variables with a missing rate below 5%, multiple imputation by chained equations (MICE) was applied. The post-imputation analysis revealed that the association between the HALP score and the outcome remained consistent with the complete-case analysis (odds ratio [OR] = 0.977, 95% CI: 0.963–0.991; complete-case OR = 0.977; see Supplementary Material Fig. 2). These findings indicate that missing data did not introduce bias and that the primary results are highly robust. Furthermore, they objectively suggest the absence of systematic differences between the eight participants with missing data and those included in the complete-case analysis.

#### Bootstrap for internal validation

To mitigate the risk of overfitting, we performed 1,000 bootstrap resampling validations. The bootstrap-corrected mean OR was 0.976 (95% CI: 0.959–0.992), which closely aligns with the original model estimate (see Supplementary Material Fig. 2). This confirms that our model exhibits strong generalization ability and stability.

## Discussion

As the population ages, the global prevalence of osteoporosis and diabetes is rising rapidly. Older postmenopausal women with T2DM face a significantly higher risk of fragility fractures due to immune-inflammatory dysregulation and inadequate nutritional reserves, posing a major public health challenge. In routine clinical practice, timely and practical assessment of their nutritional status and immune-inflammatory condition can help reduce fracture risk and improve clinical outcomes. However, the FRAX tool often underestimates the 10-year probability of fragility fractures, as it does not adequately account for these underlying factors. In this study, we investigated the relationship between the HALP score, a composite indicator of immune-inflammation and nutritional reserves, and fragility fractures. We further evaluated the performance of the HALP-adjusted FRAXplus tool in older women with T2DM.

From a pathophysiological perspective, estrogen maintains bone homeostasis by inhibiting osteoclast activity and stimulating osteoblast activity [19]. However, severe estrogen deficiency in postmenopausal women triggers a high bone turnover characterized by increased bone resorption. This leads to rapid bone mass loss and disruption of bone microarchitecture, forming the fundamental cause of postmenopausal osteoporosis and the associated increased risk of fragility fractures [20]. Notably, despite potentially exhibiting normal or even elevated BMD, individuals with T2DM face approximately double the risk of hip and non-vertebral fractures compared to age-matched peers [21]. Pathologically, advanced glycation end products (AGEs), insulin resistance, chronic inflammation, and energy deficiency may disrupt bone microarchitecture and increase bone fragility without altering bone mass, a phenomenon termed the “diabetic bone paradox (DBP)” [21]. When postmenopausal status coexists with T2DM, these mechanisms compound the effects of high bone turnover, severely compromising bone strength and resilience. This ultimately leads to a significant increase in fracture risk despite adequate BMD [22]. This is precisely why FRAX often underestimates the 10-year probability of fragility fractures, particularly hip fractures, in routine clinical practice. Therefore, it is imperative to introduce adjustment factors to optimize the FRAXplus tool and improve its predictive performance for fragility fractures.

The main findings of this study are as follows: the HALP score serves as a composite marker that effectively captures information on related immunoinflammatory and nutritional reserves. Spearman’s partial correlation analysis showed a significant negative correlation between HALP and the FRAX-estimated 10-year probability of HF, but no association with BMD or the probability of MOF. Multivariate linear regression, as a further quantitative analysis, showed that each one‑tertile decrease in HALP was associated with an approximate 0.31% increase in FRAX‑HF probability. This suggests that HALP may reflect risk factors not captured by traditional FRAX, underscoring the practical need for the novel fragility fracture risk stratification framework proposed in this study. RCS analysis demonstrated a dose-dependent relationship between HALP and fragility fractures (Z-shaped), and the 10-year probability of HF estimated by FRAX (L-shaped). Additionally, multivariable Cox regression analysis showed that fracture risk was significantly higher in the low-HALP group than in the high-HALP group. Therefore, the relationship between HALP and actual fracture risk during follow-up, as validated by RCS and multivariable Cox regression analyses, confirms HALP’s independent prognostic value. This further provides robust support for HALP as an adjustment factor for FRAXplus. Evidently, introducing the HALP score as an adjustment factor into FRAXplus may improve hip fracture risk assessment in elderly women with T2DM. As expected, this study has confirmed that after stratifying participants by HALP score tertiles, the hip fracture probability increased from an overall 2.30% (estimated by FRAX, moderate risk) to 3.00% in the low-HALP group (assessed by HALP combined with FRAX, high risk), reflecting a 30.43% increase in risk. Therefore, 27.13% of patients who initially did not meet the guideline-recommended criteria for intervention became eligible for osteoporosis treatment. This finding fully demonstrates the prognostic value of the HALP score in predicting the risk of fragility fractures.

The interactions among its four components primarily drive the HALP score’s impact on bone metabolism. The pathogenesis of anemia, such as aplastic anemia, involves immune-inflammatory crosstalk that disrupts hematopoietic stem and progenitor cells in the bone marrow, activates cytotoxic T cells and dysfunctional regulatory T cells, and induces the release of proinflammatory cytokines. Correspondingly, this leads to rapid bone loss due to osteoclast activation [23]. Furthermore, anemia impairs the blood’s oxygen-carrying capacity, leading to tissue hypoxia [24], which activates hypoxia-inducible factor-1α (HIF-1α), a key regulator of bone metabolism and a highly sensitive target of reactive oxygen species (ROS) [25]. This mechanism, in turn, enhances osteoclast activity [26]. Meanwhile, hypoalbuminemia compromises collagen synthesis and the bioavailability of insulin-like growth factor-1 (IGF-1), thereby affecting bone formation [27, 28]. Lymphocytopenia is characterized by an elevated immune-inflammatory response, leading to the release of bone-resorbing cytokines, including interleukin-1β, interleukin-6, and tumor necrosis factor-α [29–31]. Platelets contribute to thromboinflammation, with microvesicles directly promoting osteoclast differentiation [32]. Furthermore, AGEs and their receptors (RAGEs) have been implicated in driving chronic immune dysregulation in patients with diabetes [33]. The accumulation of AGEs in both cancellous and cortical bone is considered a pivotal mechanism that increases fracture risk in aging and diabetes, independent of bone mass [34]. This is consistent with the Kaplan-Meier results of this study, which indicated that lower HALP levels were associated with a higher cumulative incidence of fragility fractures. It should be noted that these interpretations of the relevant mechanisms are based on the existing literature rather than on the direct findings of this study.

In summary, the above mechanism demonstrates that HALP uniquely captures the synergistic interaction between inflammation and malnutrition in bone metabolic disorders [35, 36]. Notably, in this study, the RCS observed a dose-dependent Z-shaped relationship between HALP and fragility fractures, and an L-shaped relationship between HALP and FRAX-estimated 10-year HF probability. Combined with ROC curve analysis, these suggest a HALP threshold of 48.08 (near the ROC curve cutoff of 46.77) during the progression of metabolic bone disease. This indicates a pathophysiological critical point at which an immune-nutritional imbalance accelerates the occurrence of fragility fractures [37]. These findings support the conclusion that the HALP score is an effective FRAXplus adjustment factor, improving hip fracture risk stratification and decision-making regarding anti-osteoporosis therapy in elderly postmenopausal women with T2DM. To sum up, in routine clinical practice, for patients with FRAX-HF in the ‘moderate-risk’ range of 1.5%-3.0%, if the HALP score is < 46.77, it is recommended to upgrade them to ‘high-risk’ and consider anti-osteoporosis therapy to reduce the risk of 10-year hip fracture.

### Limitations

This study has several limitations: First, HALP was calculated from a single pre‑discharge measurement, whereas its dynamic trajectory may provide additional prognostic insight. Second, an exclusively Chinese population may introduce selection bias. Cross-ethnic validation is necessary. Third, the inherent defects of ambispective design may introduce bias. Fourth, a potential methodological limitation of this study is that its sample size was determined by data availability, as no a priori sample size calculation was conducted, although a post hoc power analysis was performed. Fourth, as a clinical observational cohort study, the findings cannot establish causality nor confirm that HALP influences fracture risk through specific biological pathways, such as IGF-1 or inflammatory cytokines. Future studies on the molecular mechanisms of fracture are necessary. Future multicentre, randomized, double-anonymized, prospective studies should be conducted to verify these findings.

## Conclusions

Our findings suggest that the HALP score may serve as an effective prognostic indicator for fragility fractures. Furthermore, the HALP score-adjusted FRAXplus tool (integrating the HALP score into FRAX) makes more elderly female patients with T2DM whose initial hip fracture probability falls below the guideline-recommended intervention threshold eligible for anti-osteoporosis therapy, thereby helping to reduce future 10-year hip fracture events.

## Supplementary Information

Below is the link to the electronic supplementary material.

Supplementary Material 1: The post-hoc power analysis for sample size estimation using G*Power software

Supplementary Material 2: Missing data handling, sensitivity analysis, and internal validation using MICE and Bootstrap

## Data Availability

All initial data from this study are available by e-mail to the corresponding author upon reasonable request.

## Abbreviations

HALP: Hemoglobin, albumin, lymphocyte, and platelet
T2DM: Type 2 diabetes mellitus
FRAX: Fracture risk assessment tool
GNRI: Geriatric Nutritional Risk Index
SII: Systemic immune-inflammation index
SAR: Systemic immune-inflammation index-to-albumin ratio
RANKL: Receptor activator of nuclear factor kappa B ligand
WHO: World Health Organization
BMD: Bone mineral density
DXA: Dual-energy X-ray absorptiometry
DR: Digital radiography
T1DM: Type 1 diabetes mellitus
LADA: Latent autoimmune diabetes in adults
TZDs: Thiazolidinediones
SGLT-2: Sodium-glucose cotransporter 2
OR: Odds ratio
HR: Hazard ratio
CI: Confidence interval
HIS: Hospital information system
aBMD: Area bone mineral density
CT: Computed tomography
MRI: Magnetic resonance imaging
MOF: Major osteoporotic fracture
HF: Hip fracture
CCA: Complete-case analysis
MICE: Multiple imputation by chained equations
SD: Standard deviations
IQR: Interquartile range
RCS: Restricted cubic spline
ROC: Receiver operating characteristic
AUC: Area under the curve
BMI: Body mass index
FT3: Free triiodothyronine
FT4: Free thyroxine
Cr: Creatinine
Ca: Calcium
25-(OH) D: 25-hydroxy vitamin D
Hb: Haemoglobin
ALB: Albumin
LS: Lumbar spine
FN: Femoral neck
TH: Total hip
DPN: Diabetic peripheral neuropathy
PVD: Peripheral vascular disease
TC: Total cholesterol
TG: Triglycerides
HDL-C: High-density lipoprotein cholesterol
LDL-C: Low-density lipoprotein cholesterol
AGEs: Advanced glycation end products
DBP: Diabetic bone paradox
HIF-1α: Hypoxia-inducible factor-1α
ROS: Reactive oxygen species
IGF-1: Insulin-like growth factor-1
RAGEs: AGEs and their receptors

## References

[1] Hong S, Saeedi P, Karuranga S, Pinkepank M, Ogurtsova K, Bruce B, Duncan, et al. IDF Diabetes atlas: global, regional and country-level diabetes prevalence estimates for 2021 and projections for 2045. Diabetes Res Clin Pract. 2022 Jan;183:109119. 10.1016/j.diabres.2021.109119. 10.1016/j.diabres.2021.109119 PMC11057359 34879977

[2] Hanna Elonheimo R, Lange H, Tolonen. Marike Kolossa-Gehring. Environmental Substances Associated with Osteoporosis-A Scoping Review. Int J Environ Res Public Health. 2021;18(2):738. 10.3390/ijerph18020738. 33467108 10.3390/ijerph18020738 PMC7830627

[3] Li Y, Teng D, Shi X, Qin G, Qin Y, Quan H, et al. Prevalence of diabetes recorded in mainland China using 2018 diagnostic criteria from the American Diabetes Association: national cross-sectional study. BMJ. 2020 Apr 28;369:m997. 10.1136/bmj.m997. 10.1136/bmj.m997 PMC7186854 32345662

[4] Zeng Q, Li N, Wang Q, Feng J, Sun D, Zhang Q, et al. The Prevalence of Osteoporosis in China, a Nationwide, Multicenter DXA Survey. J Bone Min Res. 2019;34(10):1789–97. 10.1002/jbmr.3757. 10.1002/jbmr.3757 31067339

[5] Zheng X-Q, Xu L, Huang J, Zhang C-G, Yuan W-Q. Incidence and cost of vertebral fracture in urban China: a 5-year population-based cohort study. Int J Surg. 2023;109(7):1910–8. 10.1097/JS9.0000000000000411. 37133988 10.1097/JS9.0000000000000411 PMC10389405

[6] Zoulakis M, Johansson H, Harvey NC, Axelsson KF, Litsne H, Johansson L, et al. Effect of FRAXplus adjustments on fracture risk reclassification in older Swedish women-results from the SUPERB-study. Osteoporos Int. 2025;36(9):1649–59. 10.1007/s00198-025-07588-w. 40643676 10.1007/s00198-025-07588-w PMC12460473

[7] Pan J, Xu G, Zhai Z, Wang JSQ, Huang X, et al. Geriatric nutritional risk index as a predictor for fragility fracture risk in the elderly with type 2 diabetes mellitus: A 9-year ambispective longitudinal cohort study. Clin Nutr. 2024;43(5):1125–35. 10.1016/j.clnu.2024.03.032. 38583354 10.1016/j.clnu.2024.03.032

[8] Huang D, He Q, Pan J, Zhai Z, Wang JSQ, et al. Systemic immune-inflammatory index predicts fragility fracture risk in postmenopausal anemic females with type 2 diabetes mellitus: evidence from a longitudinal cohort study. BMC Endocr Disord. 2024;24(1):256. 10.1186/s12902-024-01792-1. 39604954 10.1186/s12902-024-01792-1 PMC11600564

[9] Lu J, Wei F, Sun J, Zhai Z, Pan J, Huang S, et al. Systemic immune-inflammation index to albumin (SII/ALB) ratio as a novel dual-dimensional powerful predictor for hip fractures in elderly females with diabetes: a postmenopausal longitudinal cohort study. BMC Endocr Disord. 2025;25(1):57. 10.1186/s12902-025-01889-1. 40016771 10.1186/s12902-025-01889-1 PMC11869492

[10] Chen X, Xue L, Wang W, Chen H, Zhang W, Liu K, et al. Prognostic significance of the combination of preoperative hemoglobin, albumin, lymphocyte, and platelet in patients with gastric carcinoma: a retrospective cohort study. Oncotarget. 2015;6(38):41370–82. 10.18632/oncotarget.5629. 26497995 10.18632/oncotarget.5629 PMC4747412

[11] Xu H, Zheng X, Ai J. Lu Yang. Hemoglobin, albumin, lymphocyte, and platelet (HALP) score and cancer prognosis: a systematic review and meta-analysis of 13,110 patients. Int Immunopharmacol. 2023 Jan;114:109496. 10.1016/j.intimp.2022.109496. 10.1016/j.intimp.2022.109496 36462339

[12] Guenter Weiss T, Ganz, Lawrence T, Goodnough. Anemia of inflammation. Blood. 2019;133(1):40–50. 10.1182/blood-2018-06-856500. 30401705 10.1182/blood-2018-06-856500 PMC6536698

[13] Coin A, Perissinotto E, Enzi G, Zamboni M, Inelmen EM, Frigo AC, et al. Predictors of low bone mineral density in the elderly: the role of dietary intake, nutritional status and sarcopenia. Eur J Clin Nutr. 2008;62(6):802–9. 10.1038/sj.ejcn.1602779. 17637603 10.1038/sj.ejcn.1602779

[14] Olivier Lamy J, Everts-Graber. Elena Gonzalez Rodriguez. Denosumab for osteoporosis treatment: when, how, for whom, and for how long. A pragmatic approach. Aging Clin Exp Res. 2025;37(1):70. 10.1007/s40520-025-02991-z. 40055268 10.1007/s40520-025-02991-z PMC11889064

[15] Mark R, Thomas, Robert F, Storey. The role of platelets in inflammation. Thromb Haemost. 2015;114(3):449–. 10.1160/TH14-12-1067. 58. 26293514 10.1160/TH14-12-1067

[16] Li D-Q, Wan Q-L, Pathak JL, Li Z-B. Platelet-derived growth factor BB enhances osteoclast formation and osteoclast precursor cell chemotaxis. J Bone Min Metab. 2017;35(4):355–65. 10.1007/s00774-016-0773-8. 10.1007/s00774-016-0773-8 27628046

[17] Abdussamed Vural T, Dolanbay HY. Hemoglobin, albumin, lymphocyte and platelet (HALP) score for predicting early and late mortality in elderly patients with proximal femur fractures. PLoS ONE. 2025;20(1):e0313842. 10.1371/journal.pone.0313842. 39787124 10.1371/journal.pone.0313842 PMC11717259

[18] Alberti KG, Zimmet PZ. Definition, diagnosis and classification of diabetes mellitus and its complications. Part 1: diagnosis and classification of diabetes mellitus provisional report of a WHO consultation. Diabet Med. 1998;15(7):539–53. https://doi.org:/10.1002/(SICI)1096-9136(199807)15:7%3C539::AID-DIA668%3E3.0.CO;2-S. 10.1002/(SICI)1096-9136(199807)15:7<539::AID-DIA668>3.0.CO;2-S 9686693

[19] Cheng C-H, Chen L-R, Chen K-H. Osteoporosis Due to Hormone Imbalance: An Overview of the Effects of Estrogen Deficiency and Glucocorticoid Overuse on Bone Turnover. Int J Mol Sci. 2022;23(3):1376. 10.3390/ijms23031376. 35163300 10.3390/ijms23031376 PMC8836058

[20] Laoise MMN. Osteocytes and Estrogen Deficiency. Curr Osteoporos Rep. 2021;19(6):592–603. 10.1007/s11914-021-00702-x. 34826091 10.1007/s11914-021-00702-x

[21] Monaco MD, Bardesono CCF, Minetto MA, Busso C, Giuseppe Massazza. Prevalent vertebral fractures and the diabetic bone paradox in women who sustain a hip fracture: a cross-sectional study. Eur J Phys Rehabil Med. 2025;61(3):543–50. 10.23736/S1973-9087.25.08894-X. 40658078 10.23736/S1973-9087.25.08894-X PMC12409357

[22] Paul J, Devarapalli V, Johnson JT, Cherian KE, Jebasingh FK, Asha HS, et al. Do proximal hip geometry, trabecular microarchitecture, and prevalent vertebral fractures differ in postmenopausal women with type 2 diabetes mellitus? A cross-sectional study from a teaching hospital in southern India. Osteoporos Int. 2021;32(8):1585–93. 10.1007/s00198-021-05855-0. 33502560 10.1007/s00198-021-05855-0

[23] Mohit S, Verma V, Venkatesh S, Nityanand. Immuno-microbial crosstalk in aplastic anemia: Role of gut and viral triggers. Microb Pathog. 2026. 10.1016/j.micpath.2025.108158. Jan:210:108158. 41197919 10.1016/j.micpath.2025.108158

[24] Canan Gulmez C, Altinkaynak M, Turk N, Ozdemir OA. Hemoglobin-Inorganic Hybrid Nanoflowers with Different Metal Ions as Potential Oxygen Carrying Systems. Chem Biodivers. 2022;19(1):e202100683. 10.1002/cbdv.202100683. 34813152 10.1002/cbdv.202100683

[25] Kumari U, Ya Jun W, Huat Bay B, Lyakhovich A. Evidence of mitochondrial dysfunction and impaired ROS detoxifying machinery in Fanconi anemia cells. Oncogene. 2014;33(2):165–72. 10.1038/onc.2012.583. 23318445 10.1038/onc.2012.583

[26] Dong Wang L, Liu Z, Qu B, Zhang X, Gao W, Huang, et al. Hypoxia-inducible factor 1α enhances RANKL-induced osteoclast differentiation by upregulating the MAPK pathway. Ann Transl Med. 2022;10(22):1227. 10.21037/atm-22-4603. 36544674 10.21037/atm-22-4603 PMC9761153

[27] Ishida K, Masayoshi Yamaguchi. Albumin regulates Runx2 and alpha1 (I) collagen mRNA expression in osteoblastic cells: comparison with insulin-like growth factor-I. Int J Mol Med. 2005;16(4):689–94. 16142406

[28] Shoshana Yakar H-W, Courtland D, Clemmons. IGF-1 and bone: New discoveries from mouse models. J Bone Min Res. 2010;25(12):2543–52. 10.1002/jbmr.234. 10.1002/jbmr.234 PMC3179280 20836088

[29] Mark R, Edwards P, Sultan N, Karmali S, Ramani Moonesinghe, et al. Metabolic dysfunction in lymphocytes promotes postoperative morbidity. Clin Sci (Lond). 2015;129(5):423 – 37. 10.1042/CS20150024. 10.1042/CS20150024 25891048

[30] Yoshida H, Suzuki M, Tanaka K, Takeda S, Yogo K, Matsumoto Y. An anti-interleukin-6 receptor antibody prevents bone structural and strength loss in collagen-induced arthritis mice. Scand J Rheumatol. 2018;47(5):384–91. 10.1080/03009742.2017.1416667. 29631459 10.1080/03009742.2017.1416667

[31] Wang X, Lu L, Chen X, Liang Y. Ying Xie 1, Xijie Yu. The role and mechanism of tumor necrosis factor-alpha in alcohol-induced bone loss. Alcohol Alcohol. 2023;58(4):375–84. 10.1093/alcalc/agad026. 37092263 10.1093/alcalc/agad026

[32] Weicht B, Maitz P, Kandler B, Fischer MB, Watzek G, Gruber R. Activated platelets positively regulate RANKL-mediated osteoclast differentiation. J Cell Biochem. 2007;102(5):1300–7. 10.1002/jcb.21360. 17957725 10.1002/jcb.21360

[33] Kavitha SA, Zainab S, Muthyalaiah YS. Cordelia Mano John, Sumathy Arockiasamy. Mechanism and implications of advanced glycation end products (AGE) and its receptor RAGE axis as crucial mediators linking inflammation and obesity. Mol Biol Rep. 2025;52(1):556. 10.1007/s11033-025-10632-x. 40474023 10.1007/s11033-025-10632-x

[34] Li Z-P, Luo C, Yu X-M, Ye L-Y, Sun D, Duan C-Z, et al. Diabetic bone fragility through advanced glycation end product-collagen axis: Mechanisms and therapy of sodium glucose cotransporter 2 inhibitors. World J Diabetes. 2025;16(10):111813. 10.4239/wjd.v16.i10.111813. 41113476 10.4239/wjd.v16.i10.111813 PMC12531808

[35] Majda I, Khoury. Osteoporosis and inflammation: Cause to effect or comorbidity? Int J Rheum Dis. 2024;27(10):e15357. 10.1111/1756-185X.15357. 39352013 10.1111/1756-185X.15357

[36] Keller FSB, Gressies C, Schuetz P. Inflammation and Nutrition: Friend or Foe? Nutrients. 2023;15(5):1159. 10.3390/nu15051159. 36904164 10.3390/nu15051159 PMC10005147

[37] Geert Meermans, Jeroen C, van Egmond. Malnutrition in Older Hip Fracture Patients: Prevalence, Pathophysiology, Clinical Outcomes, and Treatment-A Systematic Review. J Clin Med. 2025;14(16):5662. 10.3390/jcm14165662. 40869488 10.3390/jcm14165662 PMC12386537

